# Biological Degradation of Spent Coffee Grounds by White Rot Fungi

**DOI:** 10.1002/mbo3.70306

**Published:** 2026-05-04

**Authors:** Anna Civzele, Anna Sila, Linda Mezule

**Affiliations:** ^1^ Water Systems and Biotechnology Institute, Faculty of Natural Sciences and Technology Riga Technical University Riga Latvia

**Keywords:** biological degradation, lignocellulose‐degrading enzymes, spent coffee grounds, waste management, white rot fungi

## Abstract

Spent coffee grounds (SCG) are extensively generated as a byproduct of coffee production and consumption. Improper disposal of SCG contributes to greenhouse gas emissions, environmental pollution, and the loss of valuable resources when landfilled or discharged into sewage systems. In response, this study investigates the biodegradation potential of SCG using selected wood‐decay fungi known for their ability to secrete a wide spectrum of lignocellulose‐degrading enzymes and degrade complex organic compounds. White rot fungi, such as *Irpex lacteus*, *Pleurotus dryinus*, and *Trametes versicolor*, were cultivated in SCG‐containing media to evaluate the degradation efficiency, fermentable sugar dynamics, and fungal enzyme secretion patterns. All tested fungi were able to metabolize SCG and exhibited active enzyme secretion during cultivation. *P. dryinus* and *T. versicolor* efficiently secreted both cellulases and laccases, with *T. versicolor* demonstrating laccase activity of 721.193 ± 41.72 U/L, indicating high oxidative potential. Fungal cultivation and enzyme production resulted in a significant carbohydrate degradation in SCG. The most significant decrease was observed in *P. dryinus*, which achieved a 43.32% reduction in SCG carbohydrates, while *T. versicolor* and *I. lacteus* ensured reductions of 39.07% and 35.55%, respectively. The findings demonstrate that SCG can serve as a low‐cost substrate for fungal enzyme production, particularly for laccase generation by *T. versicolor*, while simultaneously enabling SCG biomass degradation. Together, the study shows the potential of white rot fungi for the biological treatment of SCG, contributing to the development of more sustainable strategies for coffee waste valorization as an alternative to environmentally harmful disposal routes.

## Graphical abstract

Spent coffee grounds (SCG) are an extensively generated byproduct of coffee production and consumption. This study demonstrates the ability of white rot fungi to degrade SCG, achieving more than 40% carbohydrate reduction and producing active lignocellulose‐degrading enzymes, highlighting a potential biological approach for the valorization of this coffee waste.

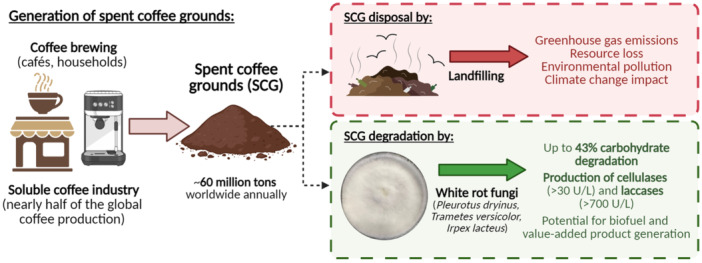

## 1 Introduction

Coffee is one of the most popular beverages, with widespread consumption across the world (Samoggia and Riedel 2019), resulting in the unavoidable generation of extensive amounts of waste by the coffee industry. Nearly half of the global coffee production is dedicated to soluble coffee preparation, with approximately 2 kg of wet spent coffee grounds generated per each kg of soluble coffee produced (Murthy and Naidu 2012). Moreover, up to 50% of the spent coffee grounds (SCG) are generated by coffee shops, restaurants, or individuals (Johnson et al. 2022), with a total annual amount estimated at 60 million tons worldwide (Forcina et al. 2023). At the same time, improper methods for disposal of this waste, such as landfilling or discharging into sewage systems, present numerous hazards and risks to human and environmental health (Fernandes et al. 2017). The decomposition of coffee waste in landfills generates greenhouse gas (GHG) that exacerbates climate change (Tapangnoi et al. 2022). Previous studies reported traditional landfilling of SCG results in approximately 612 kg CO_2_ eq emissions (Forcina et al. 2023). Moreover, landfilling SCG significantly contributes to climate change due to the high methane emissions (up to 95% of the GHG impact) (Forcina et al. 2023), which is 21 times worse than CO_2_ in its global warming potential (Roychand et al. 2023). The toxicity of SCG is also associated with its high organic content and the presence of components, such as caffeine, polyphenols, and tannins (Solomakou et al. 2022). Thus, effective treatment of coffee waste is essential for reducing its adverse environmental impacts and promoting resource efficiency and a circular economy within the coffee industry.

The common alternative to landfilling spent coffee grounds is their use as biofertilizers or soil amendments due to the high organic matter and nitrogen (1%–2.5%) (Zhu et al. 2023; Hardgrove and Livesley 2016). However, direct application of SCG has been reported to negatively impact seed germination and plant growth (Hardgrove and Livesley 2016) and is not recommended because of the toxic levels of caffeine and other organic compounds. Composting has been shown to be effective in detoxifying SCG and promoting humification, with the resulting compost demonstrating plant growth benefits (Horgan et al. 2023). Nevertheless, the application of SCG in composting can be influenced by its recalcitrant lignocellulosic components (Cervera‐Mata et al. 2022).

The majority of the waste associated with the production and consumption of coffee contains organic material and is classified as biomass (Czekała et al. 2023). The compositional analyses have shown that SCG contain hemicellulose (39%–42%), cellulose (12%–13%), lignin (23%–33%), protein (14%–18%), oil (2%–10%), and traces of ash and minerals (Ballesteros et al. 2014; Sugebo 2022; Trinh 2022; Corrado et al. 2023). Considering the content diversity and macromolecular value, SCG can be used as feedstock for both fuel and valuable molecule production (Mata et al. 2018). However, when compared to other lignocellulose‐containing biomass, such as agricultural residues, wood chips, or hay, the composition of SCG may pose challenges for biological degradation. While primarily containing polysaccharides, SCG also include a variety of phenolic chlorogenic acids and non‐phenolic acids (Abrahão et al. 2019) that can be toxic to microorganisms and inhibit microbial activity (Solomakou et al. 2022). These inhibitors make SCG more resistant to biodegradation compared to other types of lignocellulosic biomass. Moreover, since the major monosaccharides in SCG are mannose, glucose, and galactose, the use of a single enzyme in the hydrolysis of polysaccharides can limit the process efficiency (Trinh 2022).

Wood‐decay fungi that are known for their ability to secrete cellulases, hemicellulases, laccases, and peroxidases (Atiwesh et al. 2021) can be introduced as a complex tool to treat SCG. Cellulases produced by these fungi target the cellulose component of the wood, breaking it down into simpler sugar molecules such as glucose (Bhardwaj et al. 2021). Hemicellulases are secreted to degrade the hemicellulose, another major component of lignocellulose (Méndez‐Líter et al. 2021). These enzymes act synergistically to weaken the structural integrity of the lignocellulosic biomass, facilitating further degradation. Additionally, some wood‐decay fungi, such as white rot fungi, are able to produce active lignin‐modifying enzymes, such as laccases and peroxidases (Dashtban et al. 2010). These enzymes are capable of depolymerizing lignin, the complex polymer that provides rigidity and resistance to decay (Atiwesh et al. 2021), allowing full access to cellulose and hemicellulose, enabling their efficient degradation.

These processes are not only applicable to wood but can also be extended to applications for various types of organic waste materials, including SCG, that also possess the rigidity, density, and recalcitrance of lignocellulose. When applied to coffee waste biomass, wood‐decay fungi hold the potential for degradation by breaking down complex organic matter into simpler compounds, like reducing sugars. In particular, white rot fungi possess a versatile oxidative enzyme system capable of degrading and detoxifying recalcitrant compounds found in SCG. The byproducts generated from fungal treatment of spent coffee grounds can be utilized in various applications (Lee et al. 2023). Additionally, enzymes secreted by white rot fungi and extracted from fungal‐treated biomass can find further applications in biotechnological processes, such as bioremediation, wastewater treatment, and biomass bioconversion (Singh and Singh 2014).

Reflecting on challenges posed by the significant environmental impact of spent coffee grounds, this study aimed to investigate the enzymatic capabilities of *Irpex lacteus*, *Pleurotus dryinus*, *Bjerkandera adusta*, and *Trametes versicolor* to comprehensively degrade SCG biomass. For this, the selected fungi were cultivated in media containing SCG and analyzed for lignocellulose‐degrading enzyme production and carbohydrate degradation efficiency. In addition, the release of reducing sugars was determined to link polysaccharide breakdown with the release of fermentable monosaccharides and to assess the efficiency of lignocellulosic substrate conversion.

## 2 Materials and Methods

### 2.1 Microorganisms and Cultivation Conditions

White rot fungi *Irpex lacteus* DSM 9595, *Pleurotus dryinus* (Pers.) P. Kumm, *Bjerkandera adusta* DSM 23426, and *Trametes versicolor* DSM 6401 were used in the study. All cultures were maintained on potato dextrose agar (PDA) (Oxoid Ltd., Basingstoke, Hants, UK) medium at 4°C.

Prior to the experiments, each fungal species was cultivated by inoculating mycelium pieces, cut from 1 cm × 2 cm segments of agar‐grown fungal cultures, into 250 mL Erlenmeyer flasks. Each flask contained 0.8 g KH_2_PO_4_, 0.4 g K_2_HPO_4_, 0.5 g MgSO_4_·7H_2_O, 2 g NH_4_NO_3_, 2 g yeast extract, and 10 g glucose per L (pH 5.3–5.5). The cultures were incubated in an orbital shaker (New Brunswick Innova 43, Eppendorf Austria GmbH, Wien, Austria) at 30°C and 150 rpm for 96 h.

### 2.2 SCG Source and Characterization

Spent coffee grounds were collected from a coffee shop located in the campus of Riga Technical University, Latvia (56°56′59″ N 24°4′57″ E). After collection, the SCG material was stored at −20°C until further use to prevent microbial degradation of SCG. Prior to the experiments, SCG were thawed at room temperature and used directly without additional preprocessing. To determine the moisture content, SCG was dried at 105°C until constant weight. The analysis of initial SCG characteristics included the determination of total carbohydrate (40.05% ± 2.99%), moisture (66.83% ± 4.40%), and lipid content (11.14% ± 0.96%). Lignin content (25%–33% of SCG dry weight) and total phenolic content (20–30 mg gallic acid equivalents (GAE)/g dry weight) were adopted from literature sources (Corrado et al. 2023; Cho et al. 2021; Andrade et al. 2022).

### 2.3 Experimental Setup

For SCG treatment, liquid media in 250 mL Erlenmeyer flasks containing 0.8 g KH_2_PO_4_, 0.4 g K_2_HPO_4_, 0.5 g MgSO_4_·7H_2_O, 2 g NH_4_NO_3_, and 2 g yeast extract per L were supplemented with 3% w/v spent coffee grounds. Prior to experiments, all flasks were autoclaved at 121°C for 15 min. To initiate the experiments, freshly cultivated fungal cultures were inoculated into both control and SCG‐containing media at a fungal biomass‐to‐substrate dry weight (DW) ratio of 1:20 and incubated in an orbital shaker (New Brunswick Innova 43, Eppendorf Austria GmbH, Wien, Austria) at 30°C and 150 rpm for 840 h. Flasks without the addition of fungi served as a control. Liquid media samples were collected daily for fungal enzymatic activity and reducing sugar analysis. At the end of the experiments, samples of the residual SCG fraction were collected and washed with deionized water to remove fungal pellets and medium residues and used for total carbohydrate determination. Each experiment was performed in three independent replicates. All analytical measurements were conducted in triplicate.

### 2.4 Fungal Enzymatic Activity Analysis

For enzyme activity assessment, samples were first centrifuged at 8500 rcf for 5 min, and the supernatant was collected for the analysis.

Fungal cellulase activity was measured using the colorimetric CellG5 assay kit (Megazyme Ltd., Bray, Ireland) according to the protocol based on the method described by Mangan et al. (2016). The assay quantifies endo‐cellulase activity using the chromogenic substrate CellG5. In brief, 10 μL of the collected supernatant were incubated with 10 μL of CellG5 substrate solution at 40°C for 10 min. After the addition of 2% w/v Tris solution (pH 10), the absorbance of the resulting mixture was measured at 400 nm using a microplate reader (The CLARIOstar Plus, BMG Labtech, Germany). One enzyme unit was defined as the amount of enzyme required to release one micromole of 4‐nitrophenol from CellG5 per minute under the defined assay conditions.

Fungal laccase activity was determined by the ABTS method (Matsumura et al. 1987), where the oxidation of ABTS was measured by the determination of an increase in *A*_436_ (*ε*_436_ = 36,000 M^−1^·cm^−1^). The reaction mixture contained 10 mM ABTS, 0.1 M sodium acetate buffer (pH 4.5), and the supernatant of the collected sample. Absorbance was measured at 436 nm against the distilled water as a blank using a microplate reader (The CLARIOstar Plus, BMG Labtech, Germany), where one enzyme unit was defined as the amount of enzyme that is capable to oxidize one micromole of substrate per minute.

### 2.5 Total Carbohydrate Content

To determine the total carbohydrate concentration in SCG biomass before and after fungal treatment, the phenol–sulfuric acid method (DuBois et al. 1956) was used. In brief, 100 mg (DW) of SCG samples were hydrolyzed with 3% H_2_SO_4_ at 121°C for 30 min. The absorption of the resulting solution was measured at 490 nm using a UV–visible Spectrophotometer (GENESYS 150, Thermo Fisher Scientific Inc., Waltham, MA, USA).

### 2.6 Reducing Sugars

The reducing sugar analysis was performed using the dinitrosalicylic acid (DNS) method (Ghose 1987) adapted for microplate scale. In brief, 10 µL of 0.05 M sodium citrate buffer and 60 µL of 3,5‐dinitrosalicylic acid were added to 10 µL of the sample in the 96‐well microplate. All samples were heated at 100°C for 5 min. Then, 220 µL of distilled water was added to each well, and the absorption of the solution was measured at 540 nm using the microplate reader (CLARIOstar plus, BMG Labtech). The sugar concentration was defined as the mg of reducing sugars per g of dry biomass (mg/g).

### 2.7 Statistical Analysis

Data are presented as mean values ± standard deviation. Statistical differences among multiple samples were assessed using one‐way analysis of variance (ANOVA). Post hoc tests were performed using Tukey's test. An unpaired Student's *t*‐test was used to compare specific samples. A value of *p* < 0.05 was considered as statistically significant. Statistical analyses and graphical representations were performed using MATLAB (R2017a, The MathWorks, USA).

## 3 Results

### 3.1 Fungal Enzyme Activity in the Presence of SCG

**Figure 1:**
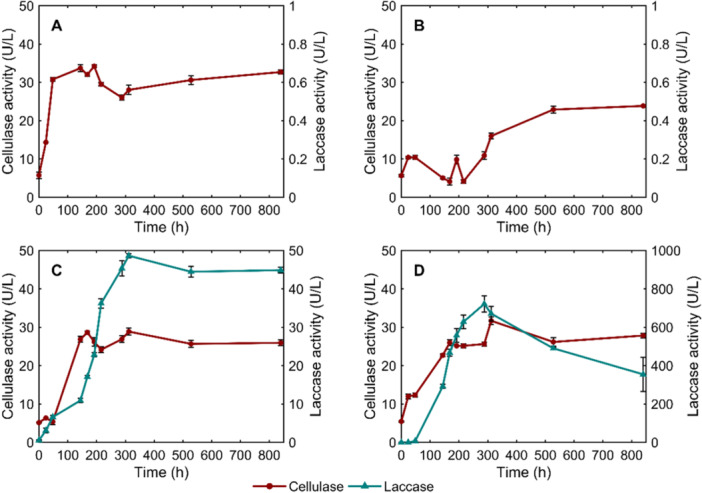
The dynamics in lignocellulolytic enzyme activity during the cultivation of (a) *I. lacteus*; (b) *B. adusta*; (c) *P. dryinus*; and (d) *T. versicolor* in SCG‐containing media.

To evaluate the biodegradation potential of selected white rot fungi, enzymatic activity was monitored throughout the cultivation period. Since SCG are composed primarily of lignocellulosic material (Zuluaga et al. 2024), cellulase and laccase, as the key enzymes involved in its breakdown, were analyzed (Figure 1).

*I. lacteus* demonstrated the most rapid and efficient cellulase activity, reaching 30.76 ± 0.12 U/L within the first 48 h of incubation. Enzyme levels were consistently maintained above 25 U/L throughout the study, reaching the highest concentration of 34.20 ± 0.44 U/L after 192 h of incubation in the presence of SCG. However, laccase activity was not detected and remained consistently absent during the experiments using *I. lacteus*. Similarly, no laccase production was identified when treating with *B. adusta*. Nevertheless, the cultivation of this white rot fungus also resulted in the cellulolytic enzyme production during the experiment. However, when compared to *I. lacteus*, the steady increase in enzyme activity in *B. adusta* appeared significantly later, after 216 h of incubation. The highest activity of cellulolytic enzymes of 15.99 ± 0.38 U/L was reached after 312 h and 22.89 ± 0.88 U/L after 528 h of incubation.

In contrast to *I. lacteus* and *B. adusta*, significantly higher laccase activity was observed in *P. dryinus* and *T. versicolor* (*p* < 0.05), with *T. versicolor* exhibiting the highest laccase productivity among all studied fungal species. In samples with *T. versicolor*, laccase activity showed a steady increase during the first 288 h, reaching the highest activity of 721.193 ± 41.72 U/L. Subsequently, laccase activity decreased to 489.85 ± 5.84 U/L after 528 h. This still remained significantly higher than the observed enzymatic activity in other fungi (*p* < 0.05). At the same time, *T. versicolor* also demonstrated consistent cellulase activity, with the first peak of 25.99 ± 0.12 U/L detected at 168 h of incubation. The highest concentration of cellulase activity (31.68 ± 0.99 U/L) was achieved after 312 h of incubation.

*P. dryinus* also demonstrated steady laccase production, achieving the highest laccase activity of 48.63 ± 0.57 U/L after 312 h. After this time point, laccase activity remained stable, and after 528 h, 44.44 ± 1.43 U/L activity was observed. Alongside the increase in laccase, this fungus also exhibited efficient cellulase production during the early stage of the experiment. Starting from 48 h, cellulase activity significantly increased from 5.38 ± 0.75 U/L to 26.84 ± 0.29 U/L at 144 h and 28.66 ± 0.35 U/L at 168 h (*p* < 0.05). The next peak and the highest activity were observed after 312 h, with cellulase activity reaching 28.86 ± 0.88 U/L and later remaining steady until the end of the experiment (25.93 ± 0.55 U/L). The presence of enzymatic activity and the consequent biomass degradation were further assessed by estimating changes in the sugar concentration and total carbohydrate content in SCG after the fungal treatment.

### 3.2 Reducing Sugar Fluctuations During SCG Treatment

**Figure 2:**
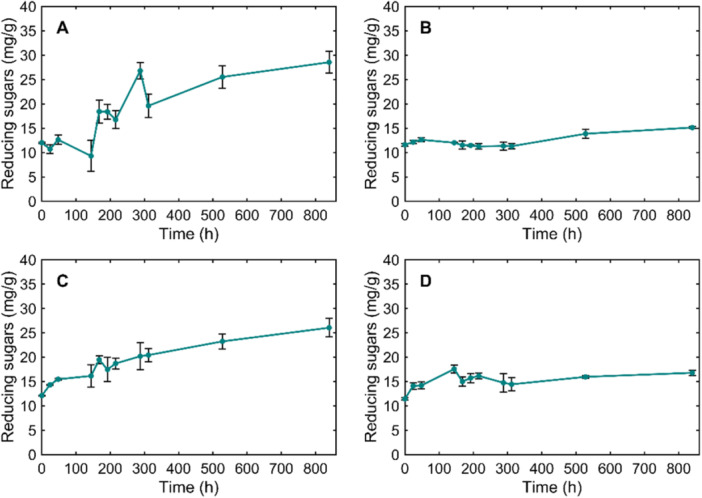
The dynamics in reducing sugar concentration during the cultivation of (a) *I. lacteus*; (b) *B. adusta*; (c) *P. dryinus*; and (d) *T. versicolor* in SCG containing media.

The high enzymatic activity in the presence of SCG was corroborated by an increase in reducing sugar concentration. The highest reducing sugar concentrations were observed in *I. lacteus* (Figure 2). The reducing sugar concentration was initially around 12.01 ± 0.15 mg/g SCG and fluctuated throughout the experiment with a steady increase over time, reaching the first peak of 26.79 ± 1.65 mg/g SCG within 288 h and the highest reducing sugar concentration (28.53 ± 2.22 mg/g) after 840 h of incubation. This constant sugar production by *I. lacteus* throughout the experiment suggests the ongoing enzymatic activity and the potential breakdown of carbohydrates.

The *P. dryinus* cultivation in SCG‐containing media also exhibited a gradual increase in reducing sugar concentrations over time. The first peak in sugar production was observed at 168 h, with a sugar concentration of 19.46 ± 0.80 mg/g. After a slight decrease, the reducing sugar production continued and peaked within 840 h of incubation, reaching similarly high sugar values to *I. lacteus* (*p* > 0.05) – 26.04 ± 1.89 mg/g biomass. *T. versicolor* exhibited a relatively stable trend in reducing sugar concentrations, with values ranging from approximately 11.42 ± 0.25 mg/g biomass initially to around 16.78 ± 0.54 mg/g biomass after 840 h of incubation. The highest sugar concentration (17.54 ± 0.78 mg/g) produced by this fungus was observed after 144 h of incubation in SCG.

Among the studied fungi, *B. adusta* exhibited the lowest reducing sugar production. Initially, the reducing sugar concentration remained relatively constant throughout the experiment, ranging from 11.24 ± 0.58 mg/g to 12.61 ± 0.49 mg/g during 312 h of the experiment. A slight increase was observed toward the later stages of incubation, with reducing sugar concentrations reaching 13.84 ± 1.01 mg/g within 528 h and 15.12 ± 0.28 mg/g biomass after 840 h.

### 3.3 Carbohydrate Degradation During SCG Treatment

The observed lignocellulose‐degrading enzyme activity of the studied fungi in SCG resulted in significant changes in the total carbohydrate content of the biomass after fungal treatment. The total carbohydrate content in untreated SCG prior to fungal inoculation was determined to be 40.05% ± 2.99% of the total dry biomass, aligning with data reported for the lignocellulosic fraction in this type of coffee waste (Bevilacqua et al. 2023). This value was used as the baseline for evaluating carbohydrate degradation. Among the studied white rot fungi, all strains demonstrated a statistically significant reduction in carbohydrate content after cultivation in SCG‐containing media (*p* < 0.05).

**Figure 3:**
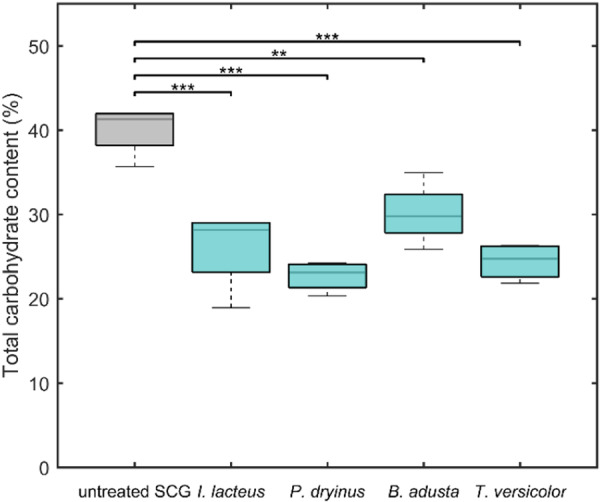
Total carbohydrate content (% of dry SCG mass) in untreated and fungal‐treated SCG biomass.

The most significant decrease was observed in *P. dryinus* (*p* < 0.05), which reduced the carbohydrate content in SCG from 40.05% ± 2.99% to 22.70% ± 1.78%, corresponding to a 43.32% reduction relative to the untreated SCG biomass (Figure 3). *T. versicolor* and *I. lacteus* exhibited similarly high degradation efficiencies, lowering the carbohydrate content to 24.41% ± 2.18% and 25.81% ± 5.31% representing 39.07% and 35.55% reductions, respectively. *B. adusta* showed the lowest, though still statistically significant reduction (*p* < 0.05) among the tested fungi, with a final carbohydrate content of 30.09% ± 3.72% of the total dry biomass, corresponding to a 24.87% reduction compared to untreated SCG.

In terms of degradation efficiency, the reduction in total carbohydrate content in SCG by *B. adusta* was significantly lower than the observed efficiency of *P*. *dryinus* (*p* < 0.05). Moreover, the performance of *B. adusta* also differed significantly from *T. versicolor* (*p* < 0.05), whereas its difference from *I. lacteus* was not statistically significant (*p* > 0.05).

## 4 Discussion

The observed degradation efficiency and high fungal enzyme activity demonstrated the capacity of all tested fungi to actively metabolize SCG biomass and indicated that these fungi not only utilized simple sugars present in SCG‐containing media but also successfully initiated the breakdown of more recalcitrant polysaccharides. However, the enzymatic activity profiles observed during the fungal treatment of SCG revealed variable strategies among the studied fungi.

The rapid and sustained cellulase activity of *I. lacteus*, reaching 30.76 ± 0.12 U/L already within 48 h, suggests an early activation of hydrolytic mechanisms targeting cellulose structures in SCG. This was reflected in the significant reduction of total carbohydrates by 35.55%, suggesting that *I. lacteus* actively degraded structural polysaccharides, releasing reducing sugars. This cellulolytic activity could be efficient in substrates where cellulose is accessible or partially exposed due to structural heterogeneity of the substrate or prior biomass treatment.

In contrast to the primarily cellulolytic strategy observed in *I. lacteus*, both *T. versicolor* and *P. dryinus* demonstrated the simultaneous production of cellulases and laccases. While *T. versicolor* exhibited a slower yet sustained cellulase production, the laccase activity reached up to 700 U/L at 288 h, far surpassing the activity of other fungal strains (*p* < 0.05). Laccases are known to oxidize phenolic structures and modify lignin‐associated components within lignocellulosic matrices (Agustin et al. 2021), which may change substrate structure and increase accessibility of carbohydrate fractions to hydrolytic enzymes. Subsequently, cellulases can hydrolyze exposed cellulose chains into soluble sugars. Correspondingly, *T. versicolor* ensured a significant carbohydrate reduction in SCG by up to 39%. Considering that spent coffee grounds are reported to contain up to 33% lignin (Corrado et al. 2023), the active production of laccases by *T. versicolor* may contribute to improved accessibility of SCG polysaccharides and higher degradation efficiency. Similarly, *P. dryinus* demonstrated the production of both cellulases and laccases, with laccase levels exceeding 48 U/L and early, steadily increasing cellulolytic activity. This resulted in the most significant total carbohydrate loss among the fungi tested (*p* < 0.05), reducing SCG carbohydrates by 43.32%. These characteristics support the potential of these fungi not only in coffee waste degradation but also in other applications requiring degradation of complex substrates and lignin‐rich materials, such as forestry residues and byproducts and lignocellulosic waste streams from various industries (Atiwesh et al. 2021). The enzymatic responses observed in *P. dryinus* and *T. versicolor* also align with previously reported findings where the studied fungi showed intense enzyme secretion when degrading complex wastewater treatment‐derived waste (Civzele et al. 2025). Both fungi demonstrated significant ligninolytic and cellulolytic enzyme activities, confirming the ability to maintain their characteristics across different lignocellulosic wastes. The potential of *T. versicolor* in biomass treatment and lignin‐degrading enzyme production was also previously demonstrated in pilot‐scale studies involving various substrates, such as oak sawdust, coffee husk, and corn bran (Montoya et al. 2021).

At the same time, *B. adusta* displayed a slower and less intense enzymatic profile. Cellulase activity exceeded 20 U/L only after 528 h, while no laccase activity was detected throughout the cultivation period. This enzyme activity pattern correlates with a comparatively moderate carbohydrate degradation (24.87% reduction), which was significantly lower than achieved by the other fungal strains (*p* < 0.05). Moreover, the comparatively low enzymatic activity in this fungus was reflected in the least pronounced reducing sugar fluctuations in SCG media during the cultivation period. This suggests that *B. adusta* may engage in slower or more selective carbohydrate consumption, possibly targeting accessible or soluble sugar fractions rather than extensively degrading polysaccharides in the first stages of cultivation. Additionally, its metabolic and enzymatic performance could have been influenced by the inhibitory compounds present in coffee waste biomass. SCG have been reported to exhibit preservative‐like properties due to their antioxidant and antimicrobial components, which can suppress fungal activity (Barbero‐López et al. 2018; Calheiros et al. 2023). Therefore, the comparatively low performance of *B. adusta* in SCG media indicates that this fungus may be more prone to SCG inhibitory effects than the other tested strains and potentially less tolerant of non‐optimal conditions. These factors show the importance of selecting fungal strains with high tolerance to such inhibitory compounds to achieve efficient biological degradation of coffee waste.

Compared to conventional pretreatment methods for lignocellulosic biomass processing, fungal treatment offers a biologically driven alternative that relies on enzymatic modification of biomass. Among traditional methods, chemical methods receive the most studies due to the high efficiency and easy operation (Xie et al. 2022). Acid treatment is reported to efficiently remove hemicellulose (Chen et al. 2024); however, it can produce inhibitory by‐products that may inhibit following microbial fermentation and affect the yield and quality of the fermentation products (Van Der Pol et al. 2016). The alkaline treatment process can effectively ensure the lignin removal and improve cellulose accessibility (Chen et al. 2024) but requires harsh process conditions with high temperature or high pressure (Yu et al. 2024). The process may also require neutralization treatment to remove alkaline substances, which increases the overall treatment costs. The traditional chemical treatments are also often performed at a high liquid‐to‐solid ratio, leading to the usage of high doses of chemicals, pretreatment liquor generation, and the need for additional biomass treatment steps before anaerobic digestion (Xie et al. 2022). In contrast, fungal systems rely on naturally secreted enzymes and can adapt to substrate composition and operate under milder conditions, reducing the need for harsh chemicals and potential subsequent pollution. However, biological processes require longer treatment times compared to chemical pretreatments (Behera et al. 2014).

The observed decrease in SCG carbohydrates and high enzyme activity suggests active release of simple sugars; a significant portion of these sugars may be assimilated for fungal growth or energy production, leaving comparatively low residual sugar concentrations in the media. Without optimization, this can present a potential disadvantage for fungal treatment compared to other methods. In contrast, the application of purified or crude enzyme extracts enables a more targeted approach. Once extracted, enzymes can be dosed with precision and act on specific substrates (Wu et al. 2022) without subsequent loss of the resulting products. Enzymatic biomass degradation is highly selective and may also require a shorter application period with a higher degradation rate compared to fungal degradation (Andlar et al. 2018). Yet, enzyme‐only treatments are also characterized by limitations. The major bottleneck of industrial enzyme application is their production cost (Sakhuja et al. 2021). In addition, the efficiency of extracted enzymes may decline over time due to denaturation or inhibition by compounds present in the biomass, such as phenolics (Zhai et al. 2018), which are also found in SCG. Moreover, a single type of enzyme may not be sufficient to fully degrade complex substrates such as lignocellulose‐containing waste. In contrast, this challenge can be addressed by adaptable fungi through the co‐expression of diverse enzymatic systems.

At the same time, the use of low‐cost substrates for enzyme production has been suggested as a strategy to reduce the economic barriers associated with industrial enzyme applications (Kululo et al. 2025). Agro‐industrial waste such as SCG may represent a particularly promising substrate in this context, as it is generated in large quantities worldwide and contains significant amounts of lignocellulosic compounds. The results obtained in this study demonstrate that fungal cultivation in SCG‐containing media resulted in active production of cellulases and laccases, indicating that SCG can function not only as a waste material requiring treatment but also as a potential feedstock for fungal enzyme production. In particular, the high laccase activity observed in *T. versicolor* suggests that SCG may support the production of oxidative enzymes relevant for lignocellulosic biomass processing and environmental biotechnology. In addition, the strategy of in situ product recovery (ISPR), such as the integration of membranes or adsorbents (Salas‐Villalobos et al. 2021), can be applied to optimize the fungal treatment process and minimize sugar consumption by fungi and improve the recovery of fermentable sugars during SCG degradation. This approach can enable the separation of valuable products before their consumption, thereby enhancing the feasibility of fungal treatment when the primary objective is biofuel or biochemical production.

In summary, the tested fungi demonstrated the ability to utilize SCG and initiate the degradation of its carbohydrate fraction through enzymatic activity. The results indicate that fungal cultivation can contribute to the biological conversion of SCG and support its potential use as a substrate for enzyme production. Such approaches may provide an alternative scenario for the valorization of coffee waste and its diversion from environmentally harmful disposal routes. Future studies could also explore integrated approaches, where fungal cultivation could be used for initial SCG pretreatment, followed by targeted enzyme application for saccharification, thereby combining the strengths of both methods.

## 5 Conclusions

The study demonstrates the potential of white rot fungi for the biological treatment of spent coffee grounds. The selection of fungal species was determined to be a crucial factor, as the studied fungi exhibited varying adaptability, enzymatic strategies, and efficiencies in degrading SCG. *I. lacteus* and *B. adusta* were primarily characterized by cellulolytic activity, while limited laccase activity was observed. Nevertheless, *I. lacteus* showed the most efficient and rapid cellulase production in SCG‐containing media, reaching enzyme activities above 30 U/L. *P. dryinus* and *T. versicolor* were observed to secrete both cellulases and laccases, indicating a broader enzymatic response to SCG. *P. dryinus* also exhibited the highest SCG degradation efficiency, reducing carbohydrate content by more than 40%. At the same time, *T. versicolor* was particularly efficient in laccase production (> 700 U/L) in the presence of SCG. While the other fungi maintained strong enzymatic performance, *B. adusta* showed greater sensitivity and lower adaptability to SCG. Overall, the study demonstrates *I. lacteus*, *P. dryinus*, and *T. versicolor* as promising candidates for the biological treatment of SCG and highlights this coffee waste as a potential substrate for the production of active lignocellulose‐degrading enzymes from white rot fungi for biotechnological applications.

## Author Contributions

**Anna Civzele:** conceptualization, formal analysis, methodology, visualization, writing – original draft, writing – review and editing. **Anna Sila:** formal analysis, investigation, writing – original draft. **Linda Mezule:** conceptualization, funding acquisition, writing – original draft, writing – review and editing.

## Ethics Statement

The authors have nothing to report.

## Conflicts of Interest

The authors declare no conflicts of interest.

## Data Availability

The data that support the findings of this study are available from the corresponding author upon reasonable request.
